# Biosynthesis of highly monodispersed, spherical gold nanoparticles of size 4–10 nm from spent cultures of *Klebsiella pneumoniae*

**DOI:** 10.1007/s13205-014-0265-2

**Published:** 2014-11-13

**Authors:** B. S. Srinath, V. Ravishankar Rai

**Affiliations:** Department of Studies in Microbiology, University of Mysore, Manasagangotri, Mysore, 570 006 Karnataka India

**Keywords:** Biosynthesis, Gold nanoparticles, Gold salt concentration, DLS, TEM

## Abstract

The development of eco-friendly approach for the preparation of monodispersed gold nanoparticles (GNPs) has received much attention for their easy application. Most of the current methods involve known protocols which employ toxic chemicals and hazardous byproducts. This greatly limits their use in biomedical fields, particularly in clinical applications. Recent research has been focused on green synthesis methods to produce different nanoparticles with suitable commercial viability. The biosynthesis of monodispersed GNPs using the spent cultures of *Klebsiella pneumoniae* as reducing and stabilizing agent has been reported. The gold salt concentration to improve monodispersity and stability of GNPs has been optimized. Synthesized GNPs were characterized by UV–Visible spectroscopy showed absorption spectra in the range of 530–560 nm at different concentrations of HAuCl_4_. At the optimum reaction concentration of 1.5 mM HAuCl_4_, absorption peak was obtained at 535 nm. The GNPs have been further characterized by X-ray diffraction, FTIR, DLS and TEM analysis. The DLS graph showed that the particles were more monodispersed. The TEM image showed the formation of spherical shaped GNPs in the range of 4–10 nm. The effect of gold salt concentration on dispersity, size and stability of the biosynthesized GNPs has been reported.

## Introduction

Nanoparticles are being synthesized by physical and chemical routes with controlled size and uniform dispersion. The chemical routes are toxic and require extreme temperature, while the physical ones are expensive and laborious. Currently, in the nanotechnology there is a growing trend to develop environmentally benign nanoparticle synthesis process that does not need any toxic chemicals. One of the important aspects in nanobiotechnology is the development of eco-friendly processes for the synthesis of stable nanoparticles, possessing well-defined shapes and uniform sizes. Biologically synthesized nanoparticles are naturally protein-capped, which prevents aggregation and avoids the use of external toxic capping agents. Microbial synthesized GNP is eco-friendly and has greater advantages over other methods since it takes place at relatively at ambient temperature and pressure (Dhas et al. 2014).

Noble metal nanoparticles such as gold, silver and platinum are widely applied in products that directly come in contact with the human body, such as cosmetic products, shampoos, detergents, soaps and toothpaste, besides clinical applications. Gold has a long history of use in medicine as red colloidal gold for revitalization in China and India (Bhattacharya and Mukherjee 2008). GNPs are biocompatible and have more applications in biology including drug delivery, labelling, photothermal therapy, tumor imaging and sensing (Noruzi et al. 2012). Synthesis of GNPs has been reported from bacteria, fungi, actinomycetes, plants and certain other biological sources. Extracellular biological synthesis of GNPs can provide an attractive and sustainable eco-friendly method for raising large quantities for advantage of easy downstream processing. Earlier studies have shown that some bacteria like *Lactobacillus* sp (Nair and Pradeep 2002), *Rhodococcus* sp (Ahmad et al. 2003)*, Pseudomonas aeroginosa* (Husseiny et al. 2007)*, Rhodopseudomonas capsulate* (He et al. 2007)*, Escherichia coli* (Du et al. 2007)*, Klebsiella pneumoniae* (Malarkodi et al. 2013) could induce GNPs synthesis.

The disadvantages with microbia-mediated nanoparticles synthesis are slow rate of synthesis and nanoparticles are mostly polydispersible. But the insights gained after optimization of synthesizing conditions such as pH, temperature and metal salt concentration and selection of microbial strain given hope in implementation of these approaches in large-scale and commercial applications. In biological methods based on plants, the size and dispersity of nanoparticles can be controlled by varying metal salts and quantity of the extracts (Khalil et al. 2012). In microbial mediated synthesis, pH plays a prominent role in controlling the particle size (Pimprikar et al. 2009). Method of varying metal salt concentration for size control has not been so for reported. Reduction of size of the GNPs has many advantages for its biological applications. These GNPs may not block the glomerulus of the kidneys as they easily pass through the urine within a short period of time when used for biological applications (Syed 2012).

In the present study, attempt has been made to obtain monodispersed nanoparticles of reduced size by optimizing the gold salt concentration. The work has focused on the development of an extracellular biosynthesis of GNP using *K. pneumoniae* and also studied optimum gold salt concentration and time required to complete the reduction. This is the first study on the effect of different gold salt concentrations on size, dispersity and stability of GNPs.

## Materials and methods

### Bacterial strain and growth conditions

The bacterial strain *K. pneumoniae* (MTCC-661) belonging to Enterobacteriaceae family as obtained from Microbial Type Culture Centre (MTCC), Chandigarh, India. The strain was sub cultured at 37 °C and stored at 4 °C for further experiments. The bacterial culture was inoculated in 250 ml flask containing 100 ml sterile Luria–Bertani (LB) broth (pH 7.0) and incubated for 24 h at 37 °C and the turbidity of the culture was adjusted to 0.5 McFarland unit (1.5 × 10^8^ cfu/ml). After incubation, the culture was centrifuged at 5000 rpm for 10 min at 4 °C to separate bacterial cells. The supernatant obtained after centrifugation was used immediately for nanoparticles synthesis.

### Biosynthesis of gold nanoparticles

The supernatant obtained from the above procedure was used for GNPs synthesis. To study the effect of gold salt concentration on biosynthesis of GNPs, experiments were carried out at different concentrations (0.3, 0.5, 1.0, 1.5, 2.0, 2.5 and 3.0 mM) of HAuCl_4_. For the synthesis of GNPs, 1 ml of spent culture (obtained by centrifugation of 1.5 × 10^8^ cfu/ml cells) was added to 4 ml of HAuCl_4_ and incubated at 37 °C for different time intervals (4, 8, 12, 24 and 28 h). The effects of gold salt concentration (HAuCl_4_) and incubation time of GNPs synthesis were studied. A cell control which lacked the gold salt and a gold salt control which lacked the bacterial metabolites were incubated with same experimental conditions. The optimum gold salt concentration and time required to complete the reactions were determined by measuring the absorbance of the resulting solutions by UV–Visible spectroscopy.

### Characterization

Reduction of metal ions was initially monitored by visual observation and further confirmed by UV–Visible spectroscopy (Thermo Scientific, Multiskan Spectrum) wave length range of 300–800 nm. The colloidal solution was added into a quartz cuvette and immediately spectral measurements were taken. The surface plasmon resonance (SPR) peaks were assessed for size and distribution of biosynthesized GNPs. The synthesized GNPs were lyophilised and dried powder was used for XRD analysis (RigakuminiFlex 11) operating at 30 kV and a current of 15 mA with Cu Kα radiation (λ = 1.5406 Å) and the 2θ scanning range was of 6–60° at 5° min^−1^. Dynamic Light Scattering (Zetasizer, Malvern) was used to determine the size distribution profile of small particles in suspension. This is the common technique followed to determine the nanoparticles size and dispersity. The morphology, dispersity and size of GNPs were analysed by TEM (Tecnai G2 spirit BioTWIN, 20–120 kv, Netherland). Sample for TEM studies was prepared by adding a drop of gold colloidal suspension onto a carbon coated 200 mesh copper grid and allowed to dry at room temperature prior to examination. Fourier transform infrared spectrophotometer (FTIR, Jasco-460 plus) was used to study the functional groups present in the biosynthesized GNPs. A small amount of dried powder was used for analysis. The spectra were recorded with wavelength range of 4,000 and 400 cm^−1^ at resolution 4 cm^−1^. The shifts in the peak maxima in different regions of the spectrum were analyzed and compared with published literature.

## Results and discussion

### Visual observations

**Fig. 1:**
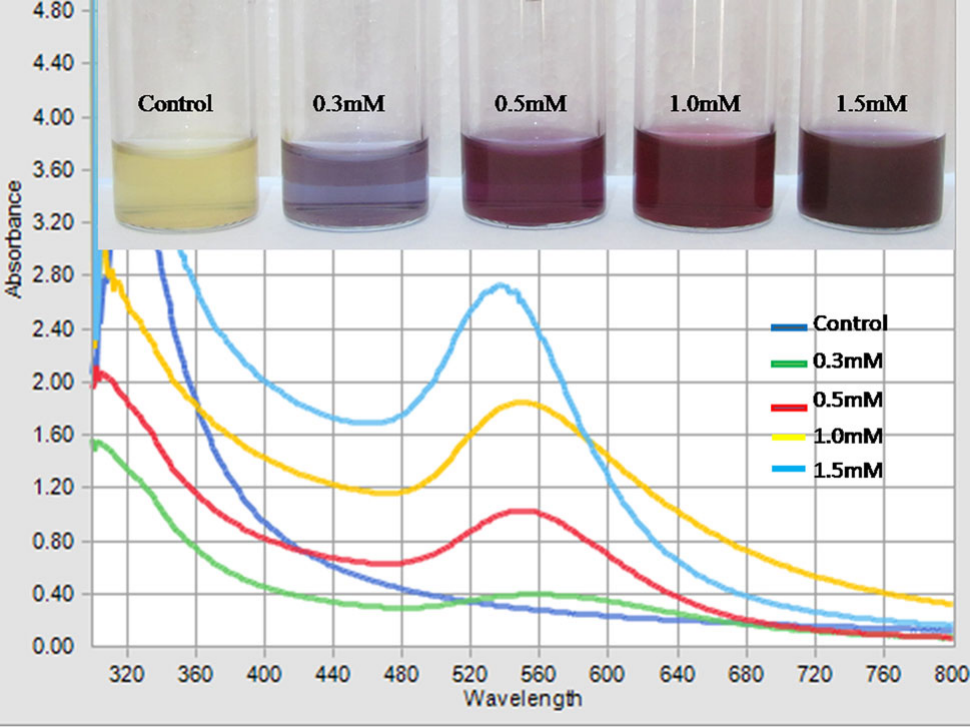
UV-Visible spectra of gold nanoparticles synthesized by spent cultures of *K. pneumoniae* at different gold salt concentrations. The inset shows photograph of gold colloids

Change of colour was perceptible in the cell free spent cultures of *K. pneumoniae*, when mixed with different concentrations of HAuCl_4_. Figure 1 shows depicts color change from pale yellow to purple in 0.3 mM and development of pink red colour is evident in 0.5, 1.0 and 1.5 concentrations of HAuCl_4_. These distinct colours are due to the variation in the size of the nanoparticles that were synthesized. Controls showed no colour change when incubated under the same conditions.

### UV–visible spectroscopy

The colloidal solution of GNPs shows intense pink color due to surface plasmon resonance (SPR) arising from collective oscillation of free conduction electrons induced by an electromagnetic field (Pereira et al. 2012). The SPR of GNPs usually has a range of 520–560 nm in aqueous solutions depending on the shape and size of the nanoparticles. With the increase in the size of the nanoparticles, the SPR shifts towards longer wavelength. The position of the absorption band mainly depends upon dielectric constant of the medium and surface-adsorbed species (Xia and Halas 2005). According to Mie’s theory, a single SPR band is expected in the absorption spectra of spherical nanoparticles, whereas anisotropic nanoparticles could give rise to two or more SPR bands depending on the shape of the nanoparticles. In the present investigation, a single SPR band was observed in each concentration of the sample that is an evidence for spherical shape of GNPs. The variations in SPR and band width of the biosynthesized GNPs are shown in Fig. 1. The color change occurred in the reaction mixture, when incubated with HAuCl_4_ at time intervals of 4, 8, 12, 24 and 28 h; maximum intensity was attained at 24 h. The peaks were obtained at wave lengths of 530–560 nm ranges. The spectrum of the reaction mixture containing 0.3, 0.5, 1.0 and 1.5 mM of HAuCl_4_ showed peaks in varied range and intensity. The reaction mixture containing 0.3 mM of HAuCl_4_ showed a broad peak in the range of 520–600 nm, which is the indication for the poly-dispersity of GNPs. The spectrum of 0.5, 1.0 and 1.5 mM of HAuCl_4_ show sharp peaks at 550, 555 and 535 nm, respectively. The sharp and intense peak observed with 1.5 mM indicates the monodispersed nature of the synthesized GNPs. Hence it is considered as the optimum salt concentration for GNPs formation. Concentrations of HAuCl_4_ above 2.0 mM (not shown in the figure) did not exhibit the peak characteristics of GNPs. Synthesis of GNPs using bacterial system has been reported by several researchers (Du et al. 2007; Husseiny et al. 2007) by using 1 mM gold salt concentration including *K. pneumoniae* (Malarkodi et al. 2013). But these studies did not provide justification for using 1 mM gold salt concentration for the synthesis. To address the issue, synthesis of GNPs by *K. pneumoniae* was monitored at different gold salt concentrations to study the effect on dispersity and size of GNPs. The result showed that reduction occurred at all concentrations up to 1.5 mM, beyond that formation of high aggregation was evident. Although reduction occurred at gold salt concentrations above 2 mM, but GNPs were not stable and aggregations were observed in the solution.

The sharp and high intensity SPR peak was observed at 1.5 mM. This could be considered as the optimum gold salt concentration for the synthesis of GNPs by *K. pneumoniae*. It is hypothesized that the biomolecules that were involved in synthesis and stabilization of GNPs work most efficiently at 1.5 mM gold salt concentration. Hence this gold salt concentration is considered optimum for GNPs synthesis by *K. pneumoniae.* The GNPs were further characterized by XRD, DLS, FTIR and TEM.

### XRD measurements

**Fig. 2:**
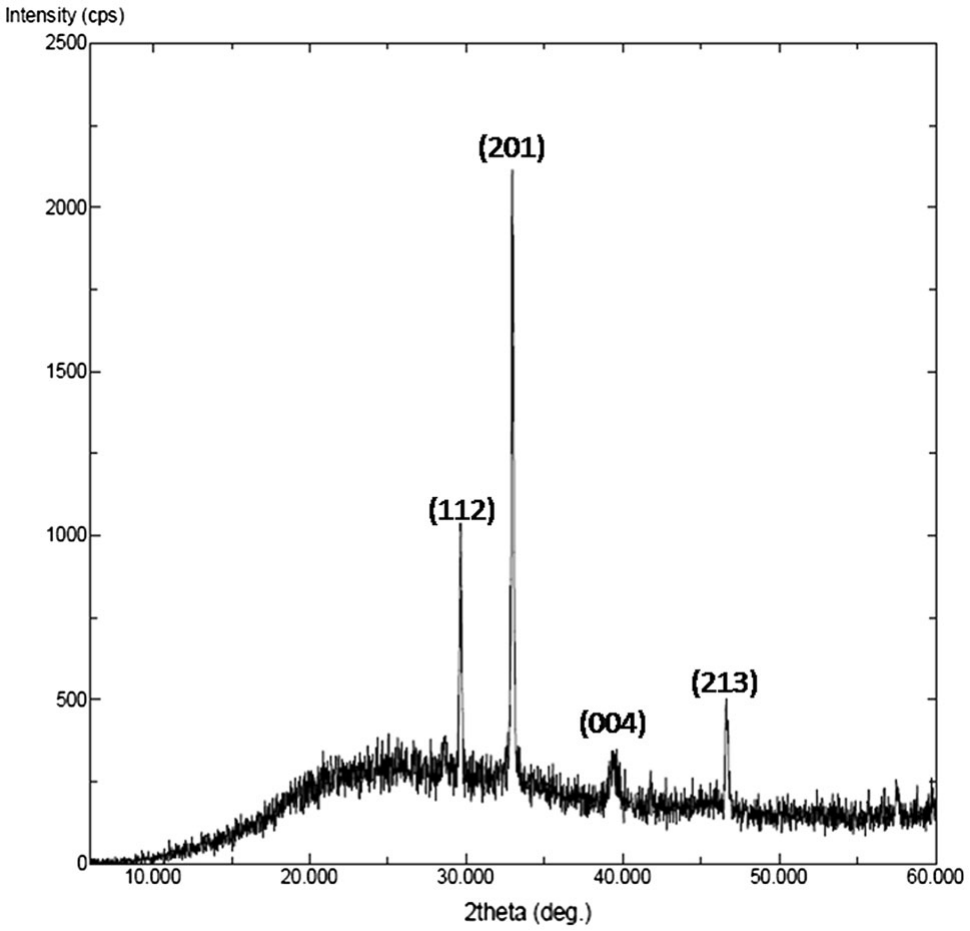
X-ray diffraction pattern of Gold nanoparticles synthesized from spent cultures of *K. pneumoniae*

X-ray diffraction measurement provides information about the crystalline nature of the synthesized GNPs. The X-ray diffraction pattern (XRD) of GNPs synthesized using spent cultures of *K. pneumoniae* is shown in Fig. 2. The strong and narrow diffraction peaks observed indicate that the product has well-defined crystalline structure. The diffraction peaks at 2*θ* values of 29.64°, 33.02°, 39.42° and 46.55° and correspond to Bragg’s reflections (112), (201), (004) and (213), respectively. The result clearly proves that GNPs formed have crystalline nature and this also agrees with earlier reports for gold nanocrystals (Karthik et al. 2013). The mean crystallite size was calculated by applying Scherr’s formula* D* = 0.94*λ*/*β*_1/2_ cos* θ*, where,* D* is the average crystal size,* λ* is the X-ray wave length (*λ* = 1.5406 Å),* θ* is Bragg’s angle (2*θ*),* β*_1/2_ is full width at half Maximum (FWHM) in radians. The mean crystallite size of nanoparticles synthesized by *K. pneumoniae* was found to be 7.20 nm and this was also further confirmed by TEM studies.

### Fourier transform infrared (FT-IR) spectroscopy

**Fig. 3:**
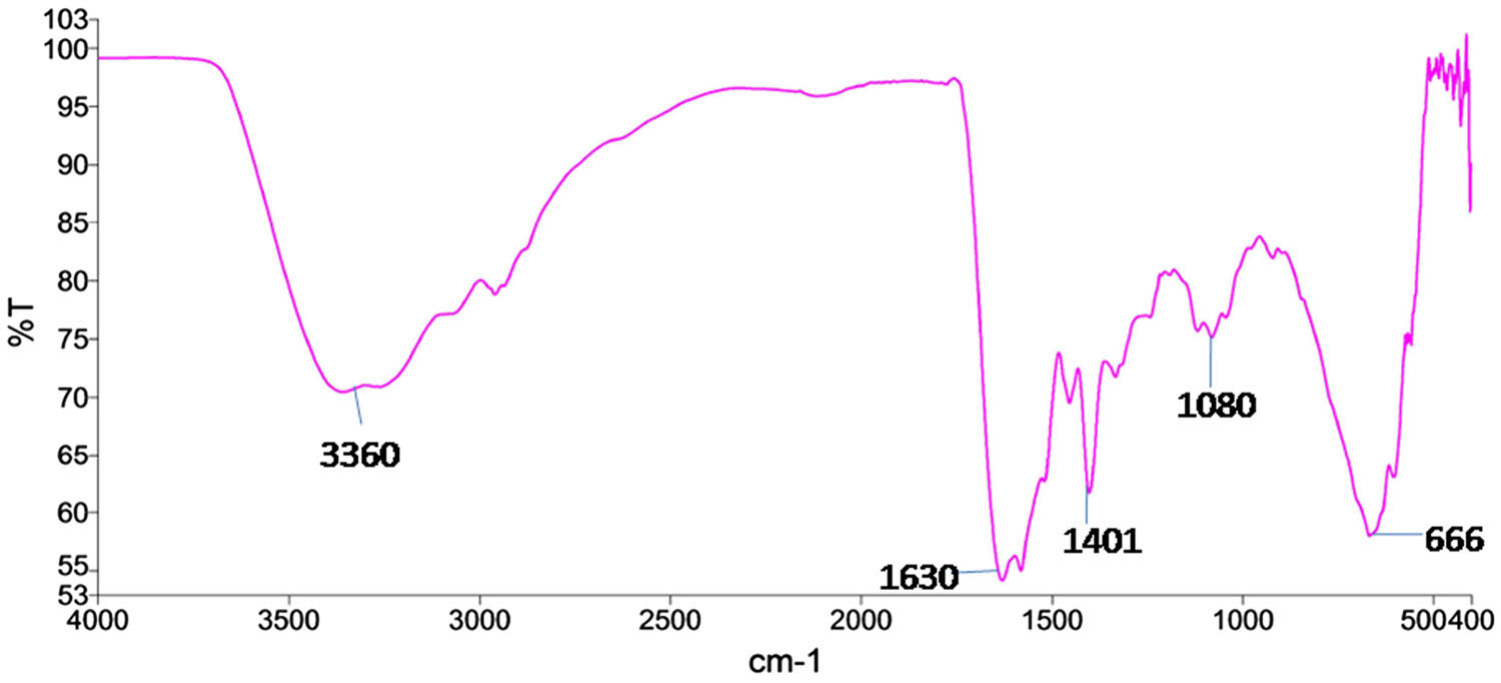
FTIR spectra of gold nanoparticles synthesized from spent cultures of *K. pneumoniae*

The identification of possible biomolecules responsible for the reduction and stabilization of GNPs can be achieved by FTIR studies. This also helps in further functionalization with other molecules for various biological applications (Suvith and Philip 2014). The FTIR spectrum reveals the presence of different functional groups as shown in Fig. 3. The IR spectra revealed strong bands at 3,360, 1,630, 1,401, 1,080 and 666 cm^−1^. The broad peak at 3,360 cm^−1^ indicates the involvement of N–H Stretch in reduction. The peak at 1630 cm^−1^ indicates the role amide I group from biomolecules, probably from proteins present in the bacterial supernatant (Das et al. 2014). The peak at 1,401 cm^−1^ signifies the presence of C–C stretch in aromatic ring. The peak at 1,080 cm^−1^ corresponds to C–O absorption (Rajesh Warluji Raut 2014) and the band at 666 cm^−1^ corresponds to alkynes. The bands observed in IR spectra of the GNPs show the presence of enzymes and protein residues on the surface of GNPs synthesized biologically. The FTIR studies proved that the carbonyl group from amino acid residues and peptides of proteins has the ability to bind metal nanoparticles and produce capped GNPs to prevent agglomeration and stabilize them in the cell-free supernatant (Rao et al. 2002). Thus, it is found that spent cultures of *K. pneumoniae* have the ability to perform dual functions of reduction and stabilisation of GNPs.

### Dynamic light scattering

**Fig. 4:**
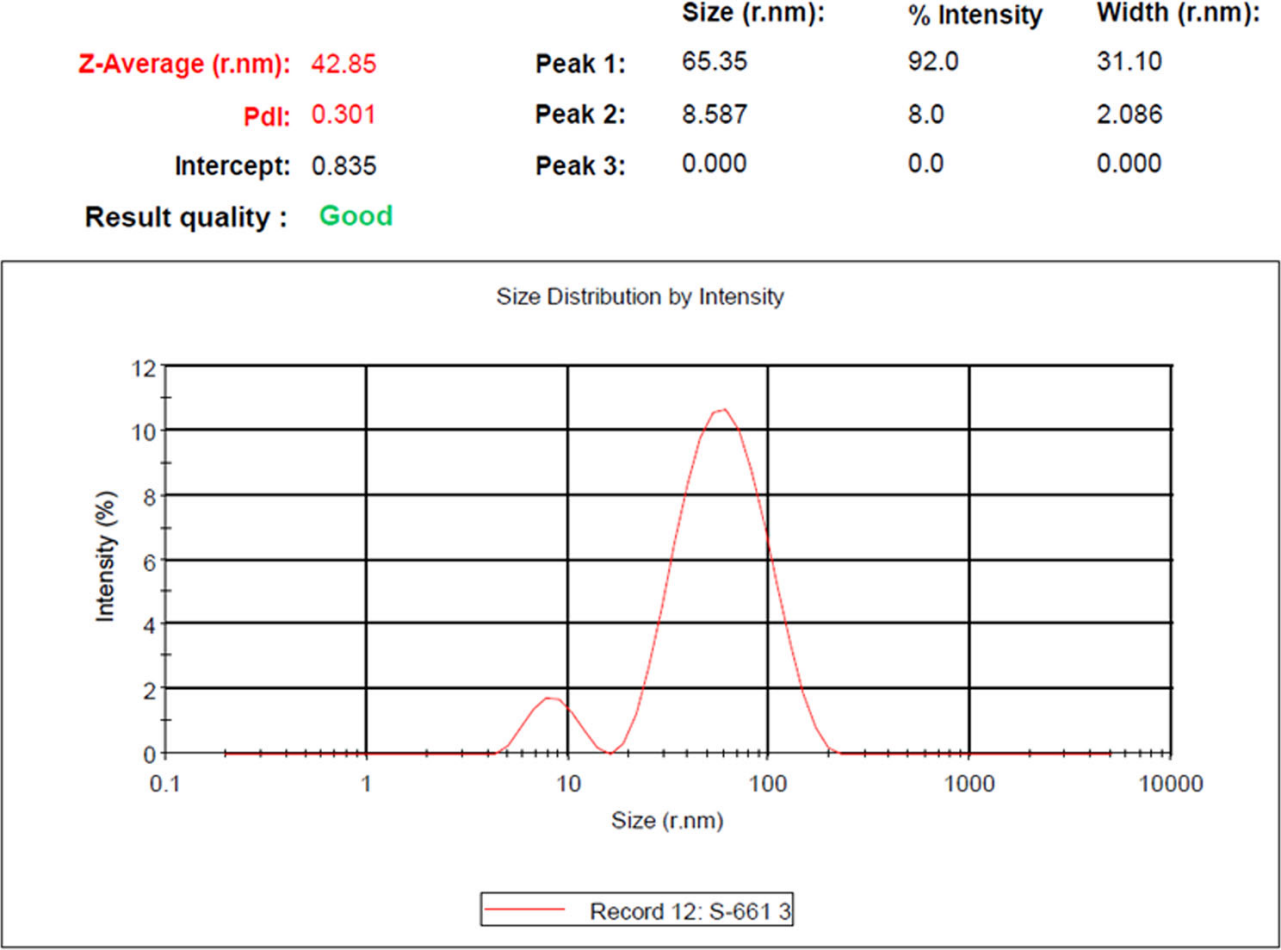
Gold nanoparticles distribution by DLS at optimised gold salt concentration (1.5 mM)

Dynamic light scattering (DLS) is used to measure the particle size in colloidal solution synthesized by different methods. The size distribution versus intensity graph is shown in Fig. 4. The average size of synthesized GNPs at 1.5 mM gold salt concentration was found to be 42.85 nm and majority of the nanoparticles have the size of 44.97 nm. The polydispersity index (PDI) was 0.301; Polydispersity index represents the ratio of particles of different sizes to total number of particles. Higher the polydispersity index less is the monodispersed particles. Synthesis of monodispersed nanoparticles by biological method has been a challenging task in nanotechnology. We discovered a method to synthesis monodispersed GNPs which involved varying of the gold salt concentration. The large size particles observed by DLS is due to the bio-organic compounds enveloping the core of the GNPs (Prathna et al. 2011).

### TEM analysis

**Fig. 5:**
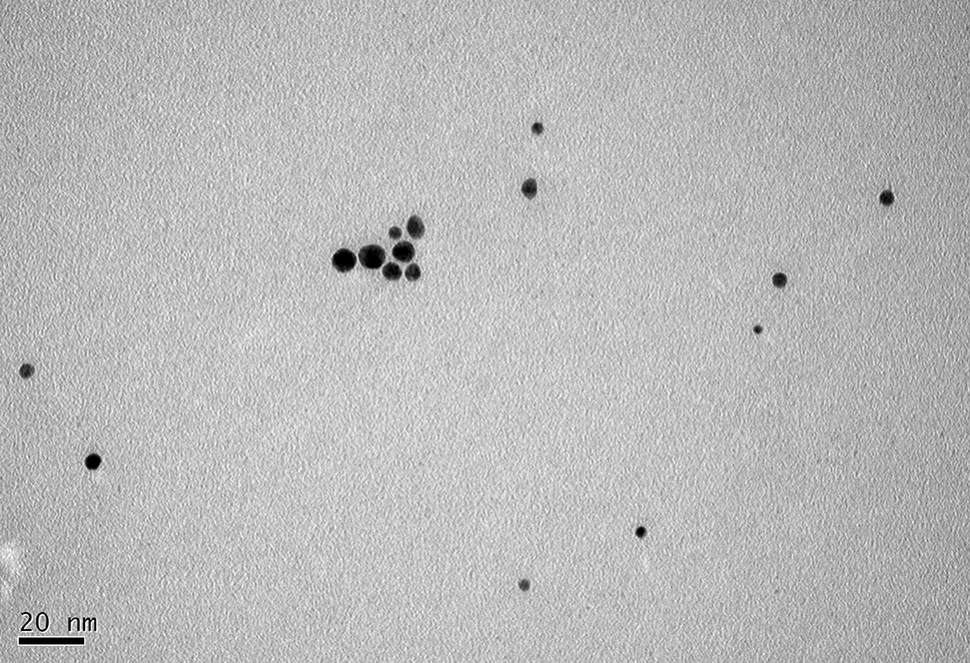
TEM image of gold nanoparticles at 1.5 mM HAuCl_4_

The result obtained from the TEM study gave a clear indication regarding the shape, size and distribution of GNPs. TheTEM image shows that, the GNPs appeared essentially spherical and reasonably monodispersed. The entire surface of the grid was uniformly spread with GNPs as shown in Fig. 5. These GNPs are monodispersed and their size range was 4–10 nm at 1.5 mM gold salt concentration. The particles were well-dispersed in solution at optimized gold salt concentration, which is due to efficient activity of both reducing and capping agents. Previous report (Malarkodi et al. 2013) showed a particle size range of 35–65 nm with polydispersity at 1 mM gold salt concentration by the same bacteria. We could get nanoparticles of reduced size (4–10 nm) with high monodispersity at optimized gold salt concentration (1.5 mM). Variations in size can also be seen in UV–vis spectra (Fig. 1) as SPR bands shift towards longer wavelengths which indicate increased size of synthesized particles (Huang et al. 2007).

## Conclusion

The investigation has shown the extracellular biosynthesis of monodispersed small sized stable GNPs using spent cultures of *K. pneumoniae*. The optimum concentration of HAuCl_4_ and maximum time required to complete the reaction were 1.5 mM and 24 h, respectively. The size, dispersity and stability of the resulting GNPs can be tuned by varying the gold ion concentration. TEM image, DLS and UV–visible spectral analysis confirmed that the GNPs synthesized at optimized gold salt concentration were monodispersed. To the best of our knowledge, this green biosynthesis method for the formation of monodispersed, small spherical GNPs of 4–10 nm size by *K. pneumoniae* is the first report of attempting to improve the monodispersity and reduced size by varying gold salt concentration. The biochemical and molecular mechanism of nanoparticles synthesis by the cell free supernatant to achieve better control over size and dispersity of nanoparticles would be explored.
